# Identification of a novel oxidative stress-related prognostic model in lung adenocarcinoma

**DOI:** 10.3389/fphar.2022.1030062

**Published:** 2022-11-18

**Authors:** Yifan Zhu, Quanying Tang, Weibo Cao, Ning Zhou, Xin Jin, Zuoqing Song, Lingling Zu, Song Xu

**Affiliations:** ^1^ Department of Lung Cancer Surgery, Tianjin, China; ^2^ Tianjin Key Laboratory of Lung Cancer Metastasis and Tumor Microenvironment, Lung Cancer Institute, Tianjin Medical University General Hospital, Tianjin, China

**Keywords:** LUAD, oxidative stress, nomogram, immune infiltration, drug response

## Abstract

**Background:** Oxidative stress (OxS) participates in a variety of biological processes, and is considered to be related to the occurrence and progression of many tumors; however, the potential diagnostic value of OxS in lung cancer remains unclear.

**Methods:** The clinicopathological and transcriptome data for lung adenocarcinoma (LUAD) were collected from TCGA and GEO database. LASSO regression was used to construct a prognostic risk model. The prognostic significance of the OxS-related genes was explored using a Kaplan-Meier plotter database. The prediction performance of the risk model was shown in both the TCGA and GSE68465 cohorts. The qRT-PCR was performed to explore the expression of genes. CCK-8, Edu and transwell assays were conducted to analyze the role of *CAT* on cell proliferation migration and invasion in lung cancer. Immune infiltration was evaluated by CIBERSORT and mutational landscape was displayed in the TCGA database. Moreover, the relationship between risk score with drug sensitivity was investigated by pRRophetic.

**Results:** We identified a prognosis related risk model based on a four OxS gene signature in LUAD, including *CYP2D6*, *FM O 3*, *CAT*, and *GAPDH*. The survival analysis and ROC curve indicated good predictive power of the model in both the TCGA and GEO cohorts. LUAD patients in the high-risk group had a shorter OS compared to the low-risk group. QRT-PCR result showed that the expression of four genes was consistent with previous analysis in cell lines. Moreover, overexpression of *CAT* could decrease the proliferation, invasion and migration of lung cancer cells. The Cox regression analysis showed that the risk score could be used as an independent prognostic factor for OS. LUAD patients in the high-risk score group exhibited a higher tumor mutation burden and risk score were closely related to tumor associated immune cell infiltration, as well as the expression of immune checkpoint molecules. Both the high- and low-risk groups have significant differences in sensitivity to some common chemotherapy drugs, such as Paclitaxel, Docetaxel, and Vinblastine, which may contribute to clinical treatment decisions.

**Conclusion:** We established a robust OxS-related prognostic model, which may contribute to individualized immunotherapeutic strategies in LUAD.

## Introduction

Lung cancer represents one of the most common malignant tumors and the leading cause of cancer-related death in the world (Siegel et al., 2016; Torre et al., 2016; Bade and Dela Cruz, 2020). Moreover, lung adenocarcinoma (LUAD) is the most common histological subtype, accounting for approximately 40% of all lung cancer types (Denisenko et al., 2018). Despite some medical treatments, the treatment of lung adenocarcinoma is not ideal due to metastasis, recurrence, or advanced stage. At present, the treatment of lung adenocarcinoma remains a significant challenge (Torre et al., 2015).

Oxidative stress is a common biochemical state, in which excessive release of reactive oxygen species (ROS) occurs to facilitate an antioxidant defense mechanism. ROS consisting of reactive nonradical species and free radicals (e.g., superoxide anion, hydrogen peroxide, singlet oxygen, *etc.*) (Lü et al., 2010). However, high levels of ROS can cause oxidative damage to proteins, lipids, and DNA (Kirtonia et al., 2020). Moreover, DNA damage plays an important role in initiating tumorigenesis. It has been reported that oxidative stress was involved in the pathogenesis of diabetes, coronary heart disease, cancer and various other diseases^[^ (Siegrist and Sies, 2017; Ighodaro, 2018; Klaunig, 2018)^]^. In the progress of tumor research, oxidative stress has been implicated in tumor cell proliferation and migration, promoting the angiogenesis of tumor cells (Sarmiento-Salinas et al., 2021). Moreover, research shows that breast cancer progression is dependent upon oxidative stress-activated stroma (Jezierska-Drutel et al., 2013). Oxidative stress has also been closely associated with a malignant phenotype of prostate cancer cells (Kumar et al., 2008). Studies have shown that exposure to inhalable mineral fiber, particulate matter smaller than 2.5 μm (PM2.5) and cigarette smoke will increase the risk of lung cancer in daily life (Pryor, 1997; Nagai and Toyokuni, 2010). Cigarette smoke can increase the level of ROS and catalyze redox reactions in lung epithelial cells. ROS can also contribute to oxidative stress and lead to both the proliferation and apoptosis of lung epithelial cells (Goldkorn et al., 2014). Moreover, another study suggests that the advanced stage of lung cancer indicates increased levels of oxidative stress (Esme et al., 2008). These studies have demonstrated that there is a close correlation between oxidative stress and LUAD progression.

The potential role of oxidative stress genes on the prognosis of lung adenocarcinoma has not been determined. In the present study, we constructed a prognostic risk score model to analyze the impact of oxidative stress on the prognosis of patients with LUAD and verified its predictive ability in The Cancer Genome Atlas (TCGA) and Gene Expression Omnibus (GEO) cohorts. Furthermore, we classified the patients into two groups and further conducted an analysis of immune cell infiltration, mutational landscape, immune checkpoints, and correlation of the drug response to explore the potential mechanisms in LUAD.

## Materials and methods

### Data collection

The transcriptome data and corresponding clinicopathological information of LUAD were collected from The Cancer Genome Atlas (TCGA) (https://portal.gdc.cancer.gov/) (Tomczak et al., 2015), comprising 535 LUAD samples and 59 normal tissues. To obtain oxidative stress-related genes accurately, 149 oxidative stress-related genes were contained from Gene Cards (https://www.genecards.org) (Safran et al., 2010) with a relevance score ≥ 16. In addition, the RNA-sequencing dataset with its clinical information downloaded from Gene Expression Omnibus (GEO) (https://www.ncbi.nlm.nih.gov/geo/) (Barrett et al., 2013) were used for validation.

### Analysis of differential expressed genes

Comprehensive analysis of oxidative stress-related genes and LUAD transcriptome data from TCGA was conducted with the help of the “limma” R package. The filter criteria were set as |Fold Change|>2, padj <0.05. Finally, 34 genes met the filter conditions. Spearman’s correlation analysis was performed to identify the correlation between screened genes.

### Construction of an oxidative stress-related risk prognostic model

Survival R package and a univariate Cox regression analysis were used to analyze the association between 34 screened genes and overall survival, respectively. The least absolute shrinkage and selection operator (LASSO) regression was conducted to construct the optimal prognostic risk model. Four OxS-related oxidative stress genes were selected. The risk score model was calculated as follows: risk score = (−0.168∗ expression value of *CYP2D6*) - (0.167 ∗ expression value of *FM O 3*)—(0.059 ∗ expression value of *CAT*) + (0.306 ∗ expression value of *GAPDH*).

### Efficacy evaluation

First, The Kaplan Meier plotter database (http://kmplot.com/analysis/) (Lánczky et al., 2016) was conducted to evaluate the association of four genes and overall survival. The samples were separated into high- and low-risk groups based on the median of risk score. The “survminer” and “survival” R Bioconductor packages were used to analyze the overall survival between the two groups using the Kaplan-Meier method with a log-rank test. The receiver operating characteristic curve (ROC) was calculated to analyze the predictive power of the risk model *via* the R “timeROC” package. Both univariate and multivariate Cox regression analysis were also performed to analyze whether the risk score and clinical factors could be independent prognostic factors for LUAD. The results were displayed in forest plots. Finally, a prognostic nomogram based on the clinical characteristics (gender, pathological stage, and age) and risk score was developed to predict the one-, two-, and three-year survival of patients with LUAD through the R “rms” package.

### Cell culture

All cell lines were obtained from the American Type Culture Collection (ATCC). BEAS-2B, H1299, and H2030 were cultured in RPMI-1640 medium with 10% fetal bovine serum. All cells were cultured in a humid environment containing with 5% CO_2_ at 37°C.

### Extraction of RNA and real-time PCR

Total RNAs were obtained from cells by using TRIzol reagent (Invitrogen). Then, cDNA was synthesized by using Prime Script RT Master Mix (TaKaRa, Dalian, China), followed by quantification by TB Green Premix Ex Taq (TaKaRa) on the 7900HT Fast Real-Time PCR System (Applied Biosystems, Foster City, CA, United States ; Thermo Fisher Scientific). The relative gene expression was calculated by using the 2^−ΔΔCT^ method. The mRNA levels were normalized by β-actin. The primer sequences were listed in Table 1.

**TABLE 1:** Sequence of primers used in this study.

Primer name	Primer sequence (5′-3′)
CYP2D6-F	TAG​TGG​TGG​CTG​ACC​TGT​TCT​CT
CYP2D6-R	TCG​TCG​ATC​TCC​TGT​TGG​ACA
CAT-F	CCA​GAA​GAA​AGC​GGT​CAA​GAA
CAT-R	GAG​ATC​CGG​ACT​GCA​CAA​AG
FMO3-F	AAT​TCG​GGC​TGT​GAT​ATT​GC
FMO3-R	TTG​AGG​AAG​GTT​CCA​AAT​CG
GAPDH-F	GGA​GCG​AGA​TCC​CTC​CAA​AAT
GAPDH-R	GGC​TGT​TGT​CAT​ACT​TCT​CAT​GG

### Transfection and overexpression vector

H1299 cells (6 × 10^5^) were seeded on each well of 6-well plates the day before transfection. According to the manufacturer’s instructions, the *CAT* overexpressing vector pcDNA-*CAT* (OE-*CAT*) and corresponding negative control (NC) were transfected into H1299 cells by Lipofectamine 2000. After 48 h, cells were harvested for total RNAs and following functional explorations.

### Proliferation assays

To evaluate the proliferation of LUAD cells, a Cell counting kit 8 (CCK-8) (Dojindo, Japan) was adopted. In short, cancer cells (5,000 cells/200 μl) were seeded into 96-well plates, and CCK-8 reagent (20 μl) was added into each well. After incubating for 2 h, the absorbance values (450 nm) were detected. For 5-ethynyl-2′-deoxyuridine (Edu) assay, cancer cells were seeded in 96-well plates, incubated with Edu (50 µM) (Sigma) for 2 h, then fixed and permeabilized. The cells were observed analyzed through a fluorescence microscopy.

### Transwell migration and invasion assays

For invasion assay, we seeded cells (8 × 10^4^) in 200 μl serum-free medium into the upper chambers coated with matrigel (BD Biosciences, San Diego, CA, United States ). Then we added 500 μl medium with 10% FBS into the lower chamber. After 24 h of incubation, the cells invading the matrigel were fixed with 4% paraformaldehyde, stained with 1% crystal violet, and imaged under a light microscope. For migration assay, cells (3 × 10^4^) were seeded into the upper chamber in a similar manner without matrigel.

### Implementation of an analysis of the level of immune infiltration

To quantify the proportions of infiltrating immune cells through the transcriptome data profiles from TCGA-LUAD patient cohort, the CIBERSORT algorithm was used to analyze the level of immune cell infiltration. The level of gene expression matrix of tumor-infiltrating immune cells was downloaded from the CIBERSORT database (https://cibersortx.stanford.edu/) (Chen et al., 2018). The CIBERSORT output of infiltrating immune cells proportion were accurate with *p* < 0.05, and only cases with *p* < 0.05 were eligible for further study. The R package “corrplot” was used to evaluate the correlation between immune cells and provide a visual representation.

### Analysis of mutational landscapes

The somatic mutation data of the LUAD patients were downloaded from TCGA. The R “maftools” package was obtained to analyze the mutation information. The Tumor Mutation Burden (TMB) considered as the mutation density of tumor genes was calculated as the transformation of total non-synonymous mutations per megabase (Schumacher et al., 2015). The difference in the TMB between the two subgroups was analyzed using a Wilcox test.

### Prediction of chemotherapeutic agents sensitivity

To explore the differences in the chemosensitivity between the low-risk and high-risk groups, The R “pRRophetic” package was used to predict half-maximal inhibitory concentration (IC50) values (Geeleher et al., 2014a; Geeleher et al., 2014b). Various common anticancer drugs and the cell line expression spectrum were downloaded from Genomics of Drug Sensitivity in Cancer (GDSC) (www.cancerrxgene.org/) (Yang et al., 2013). A ridge regression model was constructed to predict the IC50 of chemotherapy drugs.

### Statistical analysis

R software (version 4.1.3) and GraphPad Prism 9 were used for statistical analysis. Subgroup differences were analyzed by a Wilcox test. The threshold of statistical significance was set at *p* < 0.05.

## Results

### Screening of prognosis-related differentially expressed OxS genes in lung adenocarcinoma

**FIGURE 1:**
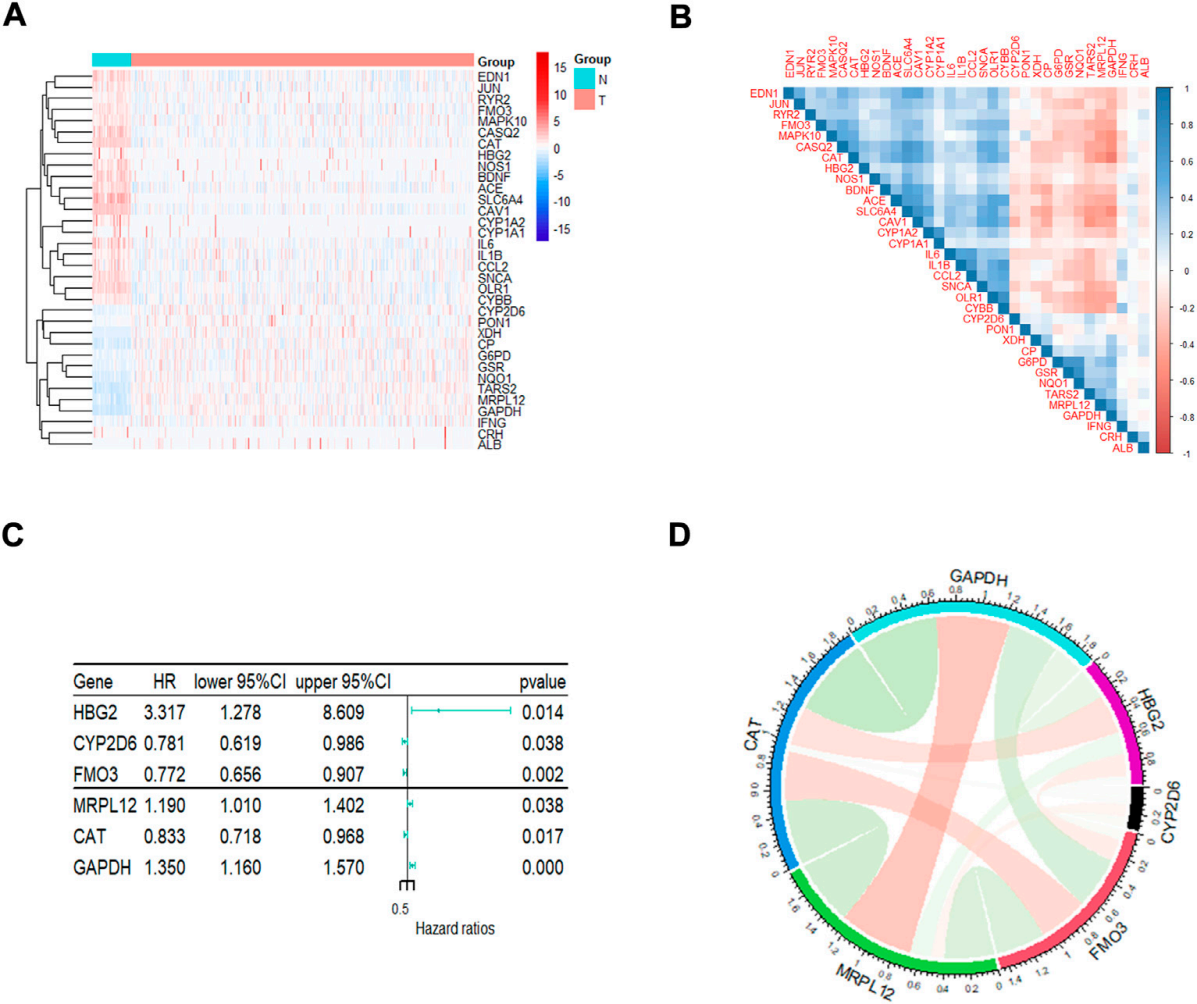
The analysis of oxidative stress genes related to prognosis in LUAD. **(A)**The heatmap visualizes the expression of oxidative stress related genes in LUAD. Blue represents low expression while red represents high expression. N represents normal samples and T represents tumor samples. **(B)** Correlation analysis of oxidative stress related genes in LUAD. **(C)** Univariate Cox regression analysis of different oxidative stress genes related to prognosis. **(D)** The correlation of screened genes from regression analysis.

A total of 148 OxS genes were included to conduct a differential expression analysis in TCGA-LUAD and the adjacent tissues. The heatmap displayed 34 differentially expressed OxS genes between the normal and tumor tissues, including 13 upregulated and 21 downregulated genes (|Fold Change|>2, padj <0.05) (Figure 1A). A correlation analysis was performed to further explore the intrinsic association between these genes. As shown in Figure 1B, the level of *CAT* expression was most negatively correlated with *GAPDH*, whereas *GSR* expression was most positively correlated with *NQ O 1*. The univariate Cox regression analysis revealed that six out of thirty-four differentially expressed genes were significantly related to OS in LUAD patients. The *HBG2* (*p* = 0.014), *MRPL12* (*p* = 0.038) and *GAPDH* (*p* = 0.000) were considered as risky genes (HR > 1), whereas the *CYP2D6* (*p* = 0.038), *FM O 3* (*p* = 0.002), and *CAT* (*p* = 0.017) were considered protective genes (HR < 1) (Figure 1C). Chord diagram of Figure 1D showed that among the six genes, the correlation between *GAPDH* and *CAT* was the most significant. The level of *GAPDH* expression was the most likely to be negatively correlated with *CAT*.

### Establishment of a prognostic risk score model based on OxS-related genes

**FIGURE 2:**
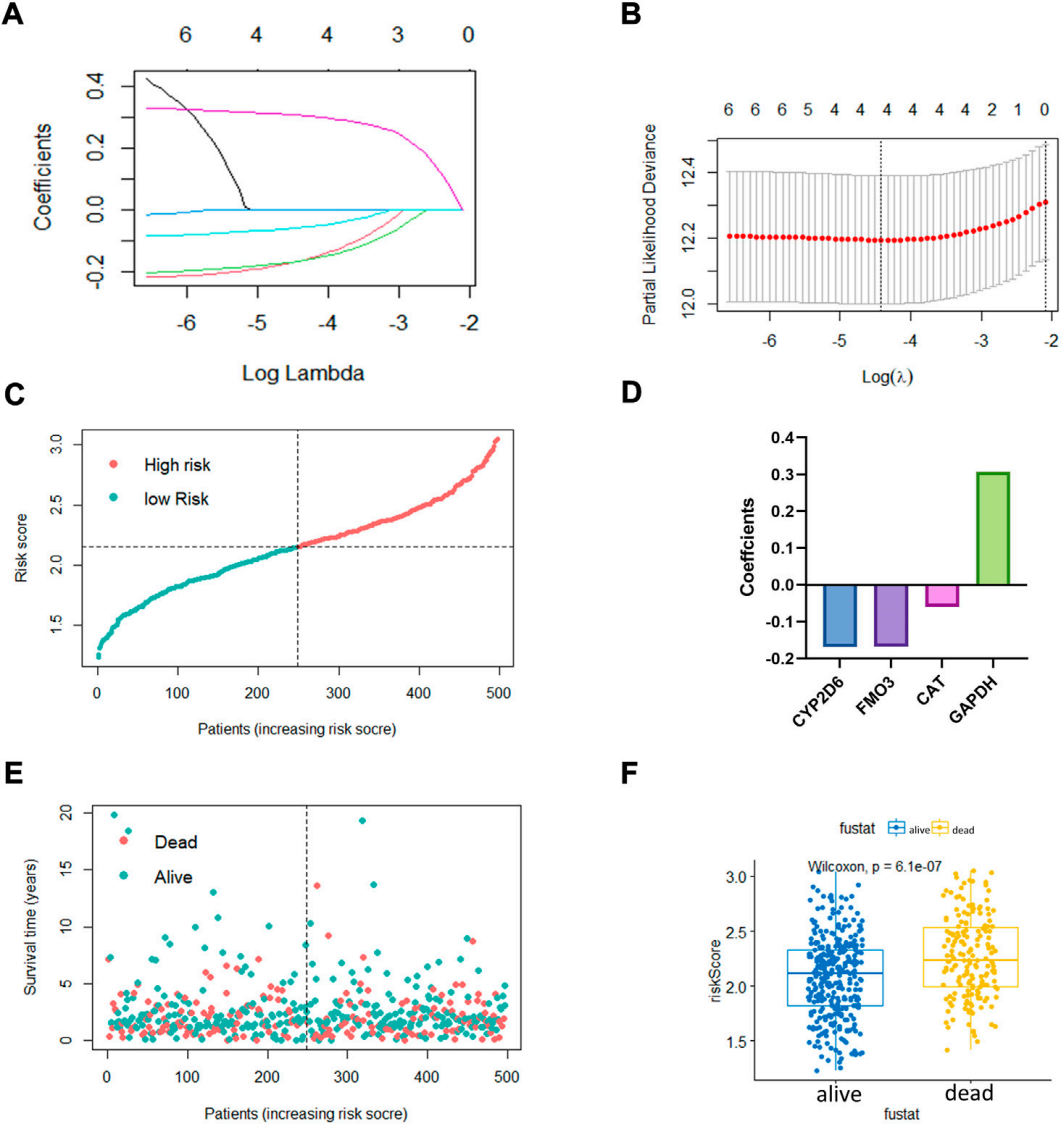
The construction of oxidative stress genes based prognostic risk model. **(A,B)** Establishment of model using least absolute shrinkage and selection operator (LASSO) Cox regression analyses. **(C)** Distribution of risk score. **(D)** Coefficients of four genes. **(E,F)** Distribution of survival status and risk score level of alive and dead group.

The LASSO regression algorithm was conducted for the OxS-related oxidative stress genes with the optimal prognostic power. Four optimal genes, *CYP2D6*, *FM O 3*, *CAT*, and *GAPDH*, were screened as factors to establish the prognostic risk model for LUAD (Figures 2A,B). The bar chart displayed the coefficients analyzed from the LASSO regression (Figure 2D). Moreover, the risk score distribution was accurate as checked in Figure 2C. The risk score level was significantly different between the survival status groups (Figures 2E,F).

**FIGURE 3:**
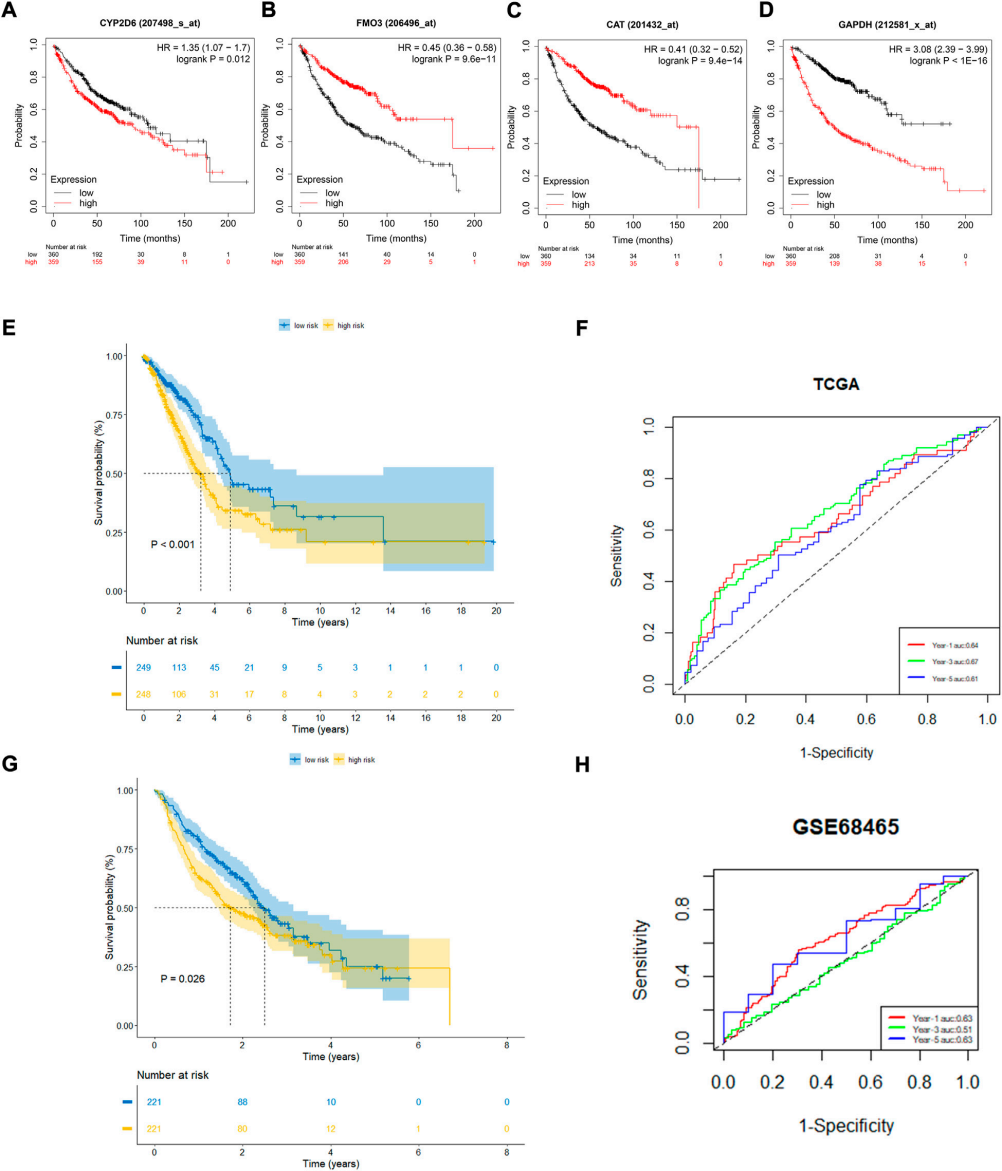
The prognostic performance of four genes and risk model. **(A–D)** Kaplan–Meier curves of four filtered genes. **(E,F)** Kaplan–Meier curves displayed that high-risk group had worse prognosis than low-risk group in TCGA cohort with 1,3,5 years AUCs were 0.64,0.67 and 0.61. **(G,H)** Kaplan–Meier curves verified in GEO cohort showed high-risk group had worse prognosis than low-risk group with 1, 3, 5 years AUCs were 0.63, 0.51, and 0.63.

To further confirm the prognostic value of the four OxS-related genes in LUAD, the correlation of gene expression with prognosis was investigated respectively in the Kaplan-Meier plotter database. The results showed the expression of *CYP2D6* (*p* = 0.012) and *GAPDH* (*P* < 1e-16) were negatively correlated with OS, whereas the expression of *FM O 3* (*p* = 9.6e-14) and *CAT* (*p* = 9.4e-14) were positively correlated with the OS (Figures 3A–D). Next, the prognostic value of the model was explored in the TCGA and GEO datasets. The risk scores of all LUAD patients in the two cohorts were acquired based on the risk calculation formula. Based on the median value of risk score, the LUAD patients in the TCGA cohort were divided into a low-risk group (*n* = 249) and high-risk group (*n* = 248). The survival analysis revealed that the patients in the high-risk group exhibited a significantly worse prognosis than the low-risk group *(p <* 0.001) (Figure 3E). The prognostic value was further verified in the GEO cohort (GSE68465). Compared with the TCGA cohort, the survival analysis in the GSE68465 cohort showed similar results to that of the OS in the high-risk group (*n* = 221) were obviously shorter than in the low-risk group (*n* = 221) (Figure 3G). The analysis indicated that the risk score model had good power for predicting the OS of LUAD patients. The AUC at 1, 3, and 5 years achieved 0.64, 0.67, 0.61, and 0.63, 0.51, 0.63, respectively for the TCGA and GSE68465 cohort (Figures 3F,H). Despite the limited 5-year AUC in the GSE68465 cohort, the model had a good ability for predicting patient survival.

### Verification of CAT functions in H1299 cell line

**FIGURE 4:**
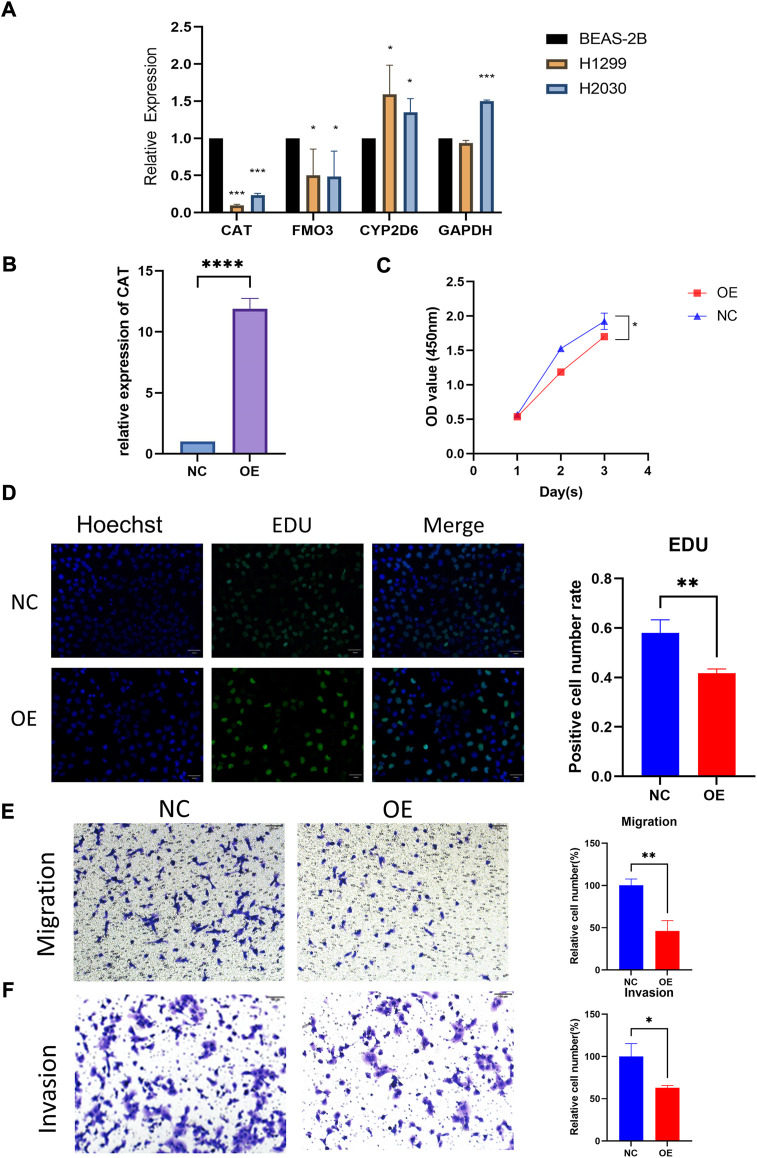
Functional verification of CAT. **(A)** Expression levels of four genes in cell lines. **(B)** Construction of CAT overexpression H1299. **(C,D)** CAT inhibited the proliferation function. **(E,F)** CAT inhibited the migration and invasion function. (*p < 0.05, **p < 0.01, ***p < 0.001, ****p < 0.0001).

The qRT-PCR assay was employed to validate the expression levels of 4 genes in cell lines. The result showed that *CAT* and *FM O 3* expressions were downregulated, whereas *CYP2D6* was upregulated in H1299 and H2030 cell lines compared with BEAS-2B. The expression of *GAPDH* was upregulated in H2030 cell line but similar in H1299 compared with BEAS-2B (Figure 4A). Then we investigated the effect of the aberrant expression of *CAT* on LUAD cells, because which possess the strongest differential expression. A gain-of-function analyses was performed *in vitro* by transfecting a vector harboring *CAT* in H1299 cells. We found that increased *CAT* expression significantly inhibited the proliferation of H1299 cells (Figures 4B,C). The similar results was also observed in Edu assays (Figure 4D). Furthermore, transwell assays with or without Matrigel results demonstrated that ectopic expression of *CAT* remarkably suppressed the invasion and migration of H1299 cells (Figures 4E,F).

### Establishment of a risk score-based prognostic nomogram for lung adenocarcinoma

**FIGURE 5:**
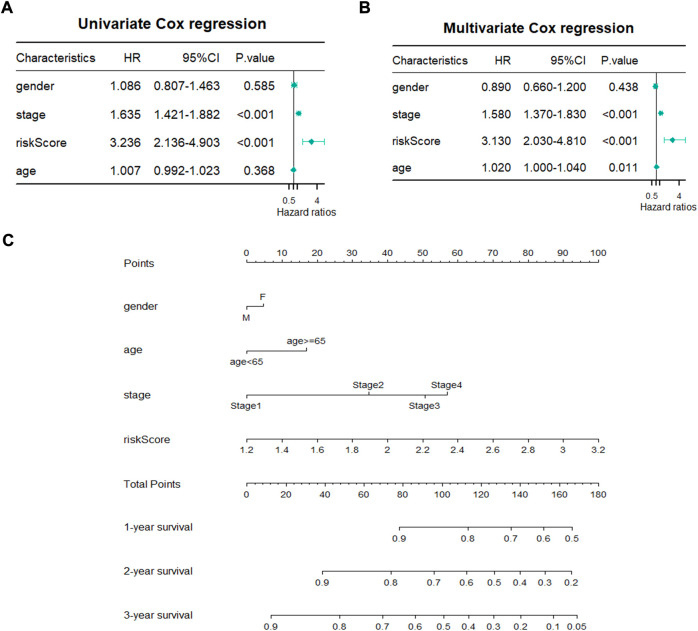
Establishment of prognostic nomogram. **(A,B)** Univariate and Multivariate Cox regression analyses of clinicopathological parameters with overall survival. **(C)** A novel prognostic nomogram based on gender, pathological stage, risk score and age.

A total of 480 cases with complete clinical information were screened for the univariate and multivariate Cox regression analysis. The univariate Cox regression analysis revealed that the OS of LUAD patients was related to stage (*p* < 0.001, HR > 1) and risk score (*p* < 0.001, HR > 1) (Figure 5A). The multivariate Cox regression demonstrated that stage (*p* < 0.001) and risk score (*p* < 0.001) could be used as independent prognostic factors for predicting the prognosis of LUAD patients (Figure 5B). Nomogram is a quantitative model for predicting patient clinical outcomes. Based on gender, age, stage, and risk score, a novel prognostic nomogram was constructed to predict the survival of LUAD patients. Each variable had its normalized corresponding point. We calculated the total points of each patient by totaling the points of all variables (Figure 5C). The 1-, 2-, and 3-year survival probabilities of the patients was estimated by drafting a vertical line from the total point axis to the survival axis, which may help clinical workers make clinical decisions.

### Differences in the level of immune cell infiltration, mutational landscapes, and immune checkpoints between the high- and low-risk groups

**FIGURE 6:**
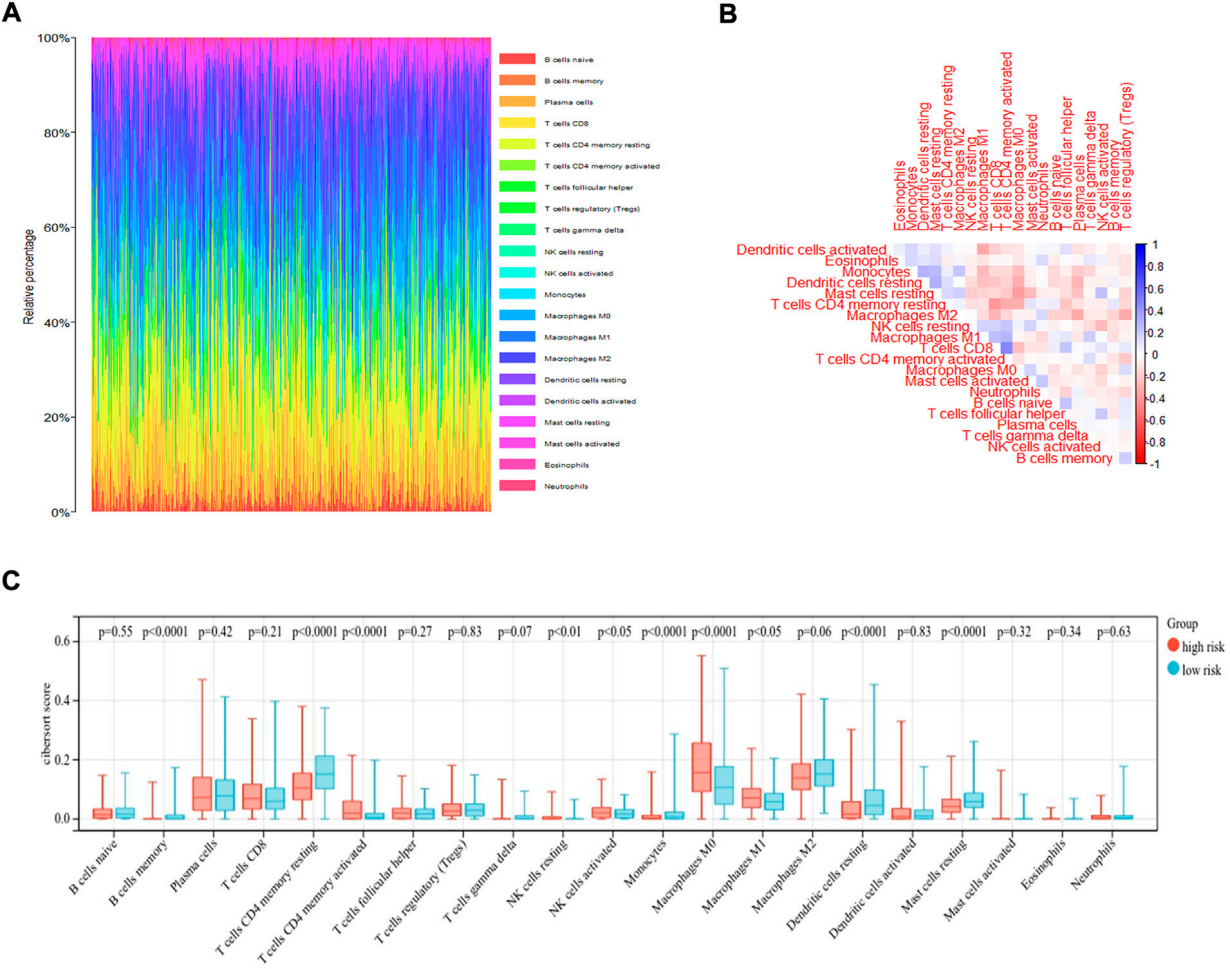
CIBERSORT analysis of TCGA-LUAD dataset. **(A)** The bar plot displayed the ratio of different immune infiltrating cells in the TCGA-LUAD datasets based on CIBERSORT algorithm. **(B)**The correlation of immune infiltrating cells. **(C)** Different kinds of immune infiltrating cells are plotted according to risk score level.

The immune microenvironment of the tumor tissue was composed of fibroblasts, stromal cells, and various immune cells, which influenced the prognosis and treatment in LUAD. To explore the correlation of risk score and level of infiltrating immune cells, CIBERSORT was used as a tool for analyzing the immune cell distribution within the TCGA-LUAD dataset. The landscape of the relative percentage of infiltrating immune cells was displayed in Figure 6A. The correlation between immune cells was analyzed in Figure 6B. All samples were divided into two groups based on the median value of the risk score. The results showed that there was a difference in various infiltrating immune cells between the two groups, including memory B cells (*p* < 0.0001), resting memory CD4 T cells (*p* < 0.0001), activated memory CD4 T cells (*p* < 0.0001), resting NK cells (*p* < 0.01), activated NK cells (*p* < 0.05), Monocytes (*p* < 0.0001), M0 macrophages (*p* < 0.0001), M1 macrophages (*p* < 0.05), resting dendritic cells (*p* < 0.0001), and resting mast cells (*p* < 0.0001) (Figure 6C). In contrast, there was no statistical significance in the number of naive B cells, plasma cells, CD8 T cells, follicular helper T cells, regulatory T cells, gamma delta T cells, M2 macrophages, activated dendritic cells, activated mast cells, eosinophils and neutrophils between the two groups. The proportion of each immune cell type was provided in Supplementary Table S2.

**FIGURE 7:**
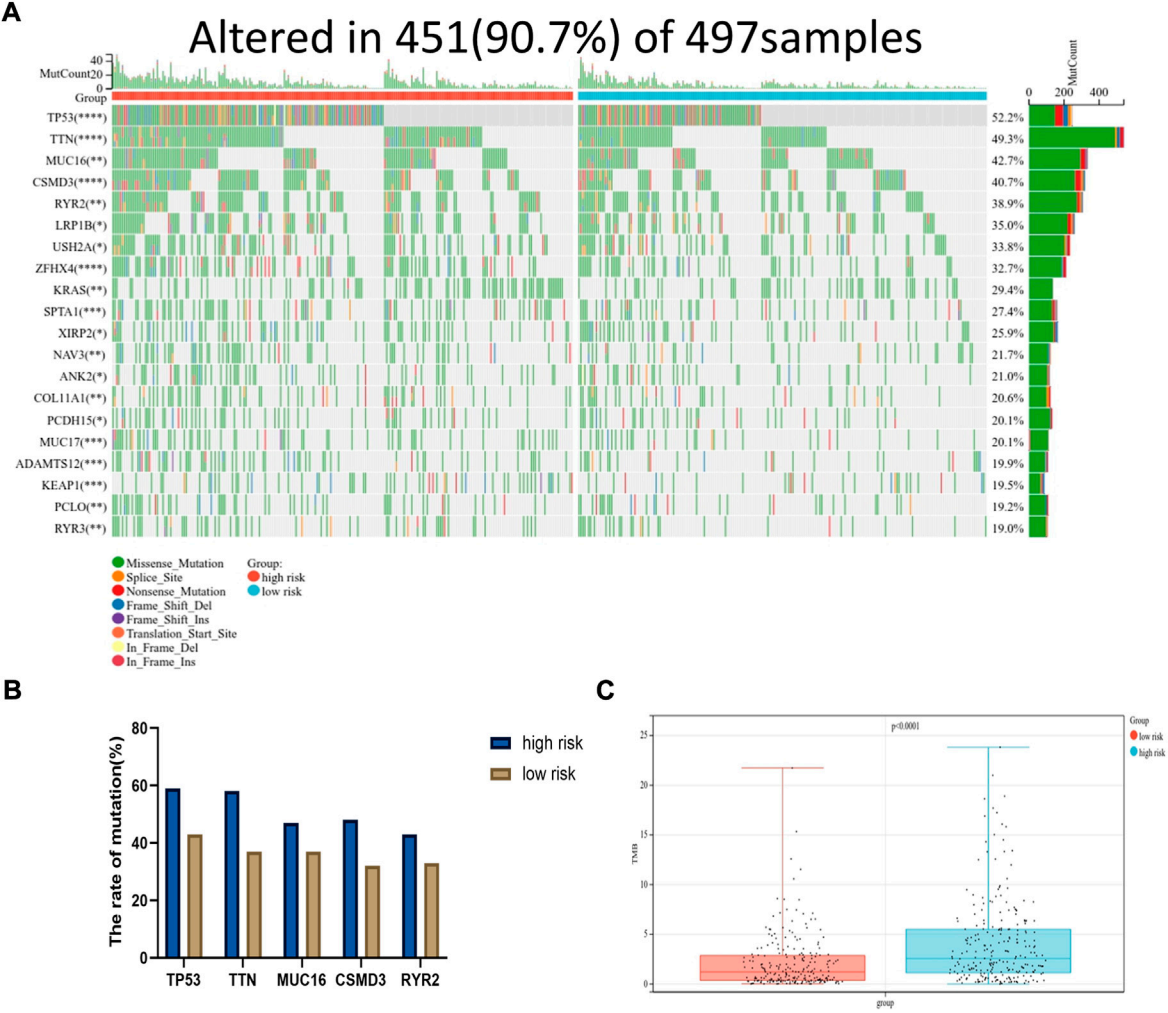
Mutational landscape displayed *via* TCGA-LUAD database. **(A)** The waterfall diagram exhibited top 20 genes with highest mutation frequency in LUAD between high and low risk group (*p < 0.05, **p < 0.01, ***p < 0.001, ****p < 0.0001). **(B)** The mutation frequency of top five genes. **(C)** The tumor mutation burden of high-risk group was significantly higher than that in low-risk group, *p* < 0.0001.

Moreover*,* the somatic mutation information of TCGA-LUAD patients was used to explore the correlation between the risk score and mutational landscape. After excluding samples with incomplete mutation information, 451 LUAD patients from the TCGA database were incorporated into the analysis. The samples were divided into a low- and high-risk group based on the median risk score. Detailed mutation information of each gene in all samples was exhibited by a waterfall plot. The top 20 mutated genes were displayed in the plot, whereas the different colors represent different mutation types. Missense mutations were the most common mutation type among the 20 genes shown. Moreover, the mutation frequency significantly differed between the low- and high-risk groups in each gene cohort. We found that more mutation events occurred in the high-risk group than in the low-risk group (Figure 7A). The tumor mutation burden (TMB) was visualized in Figure 7B. The TMB in the high-risk group was appreciably higher compared with the low-risk group, indicating the superior effect of immunotherapy. (Figure 7C)

**FIGURE 8:**
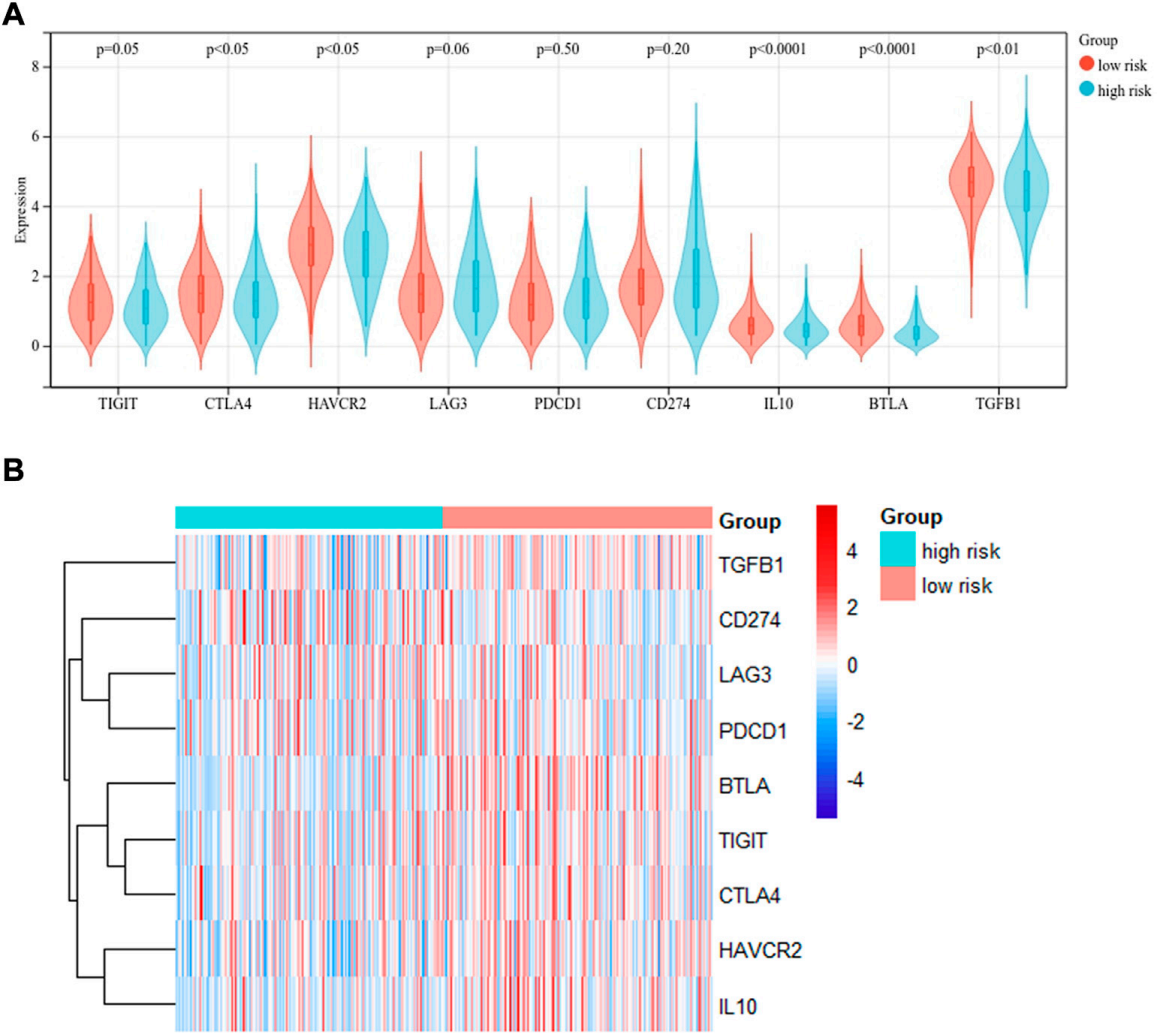
Correlation between risk group and immune checkpoints. **(A)** The difference of immune checkpoint expression between the two groups divided by risk score level. **(B)** Heatmap showed the expression of immune checkpoints in two groups.

Immune checkpoint molecules are regulatory molecules that play a role in the immune system and play a critical role in maintaining self-tolerance, preventing an autoimmune response, and controlling the timing and intensity of the immune response. The difference in the levels of immune checkpoint expression in the low- and high-risk groups was compared according to the median value. As shown in Figure 8A, the level of TIGIT (*p* = 0.05), CTLA4 (*p* < 0.05), HAVCR2 (*p* < 0.05), IL-10 (*p* < 0.0001), and TGFB1 (*p* < 0.01) differed between the two groups, whereas no statistical difference was observed in the level of LAG3, PDCD1, and CD274 between the two groups. The results suggested that the risk score may be correlated with some immune checkpoints. It may be helpful in the immunotherapy of LUAD. The distribution of immune checkpoint expression levels was also visualized by heatmap in Figure 8B.

### Therapeutic potential of the OxS-related risk score in lung adenocarcinoma

**FIGURE 9:**
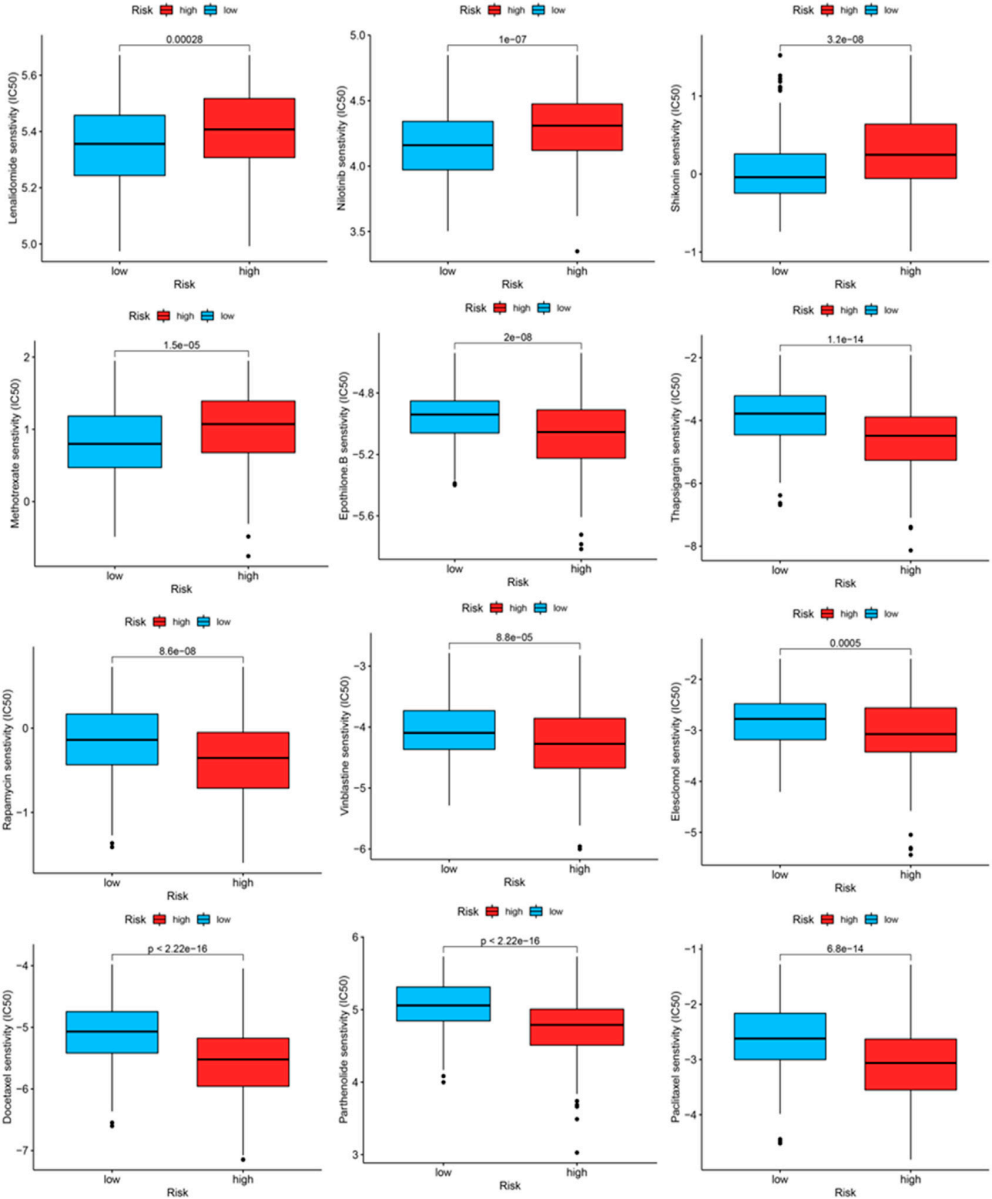
The IC50 of chemotherapeutic drugs compared between high-risk and low-risk groups.

We identified some chemotherapeutic drugs and immunosuppressors *via* pRRophetic according to the OxS-related risk score. Lenalidomide (*p* < 0.001), nilotinib (*p* < 0.001), shikonin (*p* < 0.001), methotrexate (*p* < 0.001), displayed lower sensitivity in the high-risk group, whereas epothilone (*p* < 0.001), thapsigargin (*p* < 0.001), rapamycin (*p* < 0.001), vinblastine (*p* < 0.001), elesclomol (*p* < 0.001), docetaxel (*p* < 0.001), parthenolide (*p* < 0.001), and Paclitaxel had higher sensitivity in the high-risk group (Figure 9).

## Discussion

As a substantial threat to global public health, lung cancer is associated with a poor prognosis and high mortality (Siegel et al., 2021). Thus, the prognostic gene signatures for the prognosis of LUAD are extremely important for predicting patient survival rate and drug response. Oxidative stress can cause DNA damage and further lead to tumorigenesis and progression. Therefore, the relationship between oxidative stress and its related genes and cancer has attracted substantial attention; however, the relationship between oxidative genes and LUAD has not yet been elucidated. In this study, we constructed a prognostic model based on four oxidative stress genes to predict the overall survival of LUAD patients.

A total of 148 differentially expressed oxidative stress genes were contained in the TCGA database. A univariate Cox regression analysis was used to identify the OxS-related genes and a LASSO regression analysis was conducted to shrink the range to the best. Finally, four oxidative stress genes, including *CYP2D6*, *FM O 3*, *CAT*, and *GAPDH* were identified, which were associated with the prognosis of LUAD patients. The *CYP2D6* gene polymorphisms may enhance oxidative stress and induce oxidative damage (Arafa and Atteia, 2018). Moreover, the *CYP2D6* genotypes can predict Tamoxifen discontinuation and prognosis in patients with breast cancer (He et al., 2020). *FM O 3* is an important oxidative drug metabolizing enzyme, which is Closely related to oxidative stress-responsive transcription factor *NRF2* (Klick and Hines, 2007; Rudraiah et al., 2016). Meanwhile, *FM O 3* has been reported to be investigated as a prognostic marker in hepatocellular carcinoma (Zhu et al., 2021). *CAT* is a key enzyme in the metabolism of H2O2 and play a critical role in the antioxidant defense system of cells (Nadif et al., 2005; Goyal and Basak, 2010; Glorieux and Calderon, 2017). Silenced *CAT* expression increased the susceptibility of the cancer cell line BT-20 to oxidative stress (Klingelhoeffer et al., 2012). Moreover, higher *CAT* expression in mesothelioma was associated with a better prognosis (Kahlos et al., 2001). *GAPDH* is a glycolytic enzyme which can mediate cell death under oxidative stress (Nakajima et al., 2009; Nakajima et al., 2017). Previous studies showed that *GAPDH* was involved in apoptosis, the maintenance of DNA integrity, and tumor angiogenesis (Sirover, 2018). Moreover, the level of *GAPDH* expression was up-regulated in human colorectal carcinoma tissues compared with the normal adjacent tissues, and the level of *GAPDH* expression was also increased in colon cancer cell lines (Tang et al., 2012). These results suggest that the four genes may also play an important role in the tumorigenesis and progression of LUAD.

In addition, a novel prognostic model was constructed based on the four screened genes. To the best of our knowledge, our research is the first to build an oxidative stress related prognostic model for LUAD. In addition, the model was confirmed to be an independent prognostic factor for LUAD according to the univariate and multivariate Cox regression. The prognostic value for predicting LUAD patient prognosis was identified with a survival analysis and time ROC analysis. A predictive nomogram based on a signature was constructed to predict the clinical outcomes of LUAD patients.

The proportion of infiltrating immune cells plays a significant role in the response to immunotherapy and cancer progression. Immune regulation is recognized to be associated with the prognosis of patients (Efremova et al., 2018). We verified patients in the low-risk and high-risk groups according to the model. Thus, results showed that patients in the high-risk group had higher NK cell infiltration and a lower level of mast cells than patients in the low-risk group. According to the tumor immunoediting hypothesis (Hellmann et al., 2018), the patients in the high-risk group have higher immunosuppression but lower immunoreactivity than the low-risk group. The number of M0 macrophages in patients in the high-risk group was higher than in the low-risk group. A higher proportion of M0 macrophages was associated with a worse patient prognosis (Farha et al., 2020). The difference in the infiltrating microenvironment may contribute to cancer progression and lead to a poorer prognosis. The differential expression of immune checkpoints between the two groups suggested the different effects of immunotherapy (Hiam-Galvez et al., 2021). In our study, LUAD patients in the high-risk group were found to exhibit higher levels of TMB. TMB in the tumors was associated with the objective response and may predict the survival of patients according to recent studies (Rizvi et al., 2015; Samstein et al., 2019). The transcriptome data from TCGA were contained to explore the sensitivity of patients to antineoplastic drugs between the two groups. The high-risk group was more sensitive to epothilone, thapsigargin, rapamycin, vinblastine, elesclomol, docetaxel, parthenolide and paclitaxel while displayed lower sensitivity to lenalidomide, nilotinib, shikonin and methotrexate. Our study revealed the sensitivity of patients to antitumor drugs as verified by risk groups, which may provide a direction for researchers to develop treatment programs with high efficacy.

However, this study has several limitations which must be considered. In our study, the risk model and correlated nomogram were constructed using data from the TCGA and GEO databases which have great robustness. However, caution should be exercised if extrapolating the results of our study to ethnicities other than Asians or Whites. Thus, this study requires further experimental studies.

In conclusion, this study constructed a novel oxidative stress gene-related model comprising *CYP2D6*, *FM O 3*, *CAT*, and *GAPDH* to predict the prognosis of LUAD patients. A significant difference was observed between the high- and low-risk groups in the immune cell infiltration, levels of TMB and immune checkpoint expression. In addition, the model could reveal the sensitivity to chemotherapeutic drugs. Furthermore, the model can help researchers understand the correlation between oxidative stress and LUAD and may also provide novel insight for future anti-tumor immunotherapy.

## Data Availability

Publicly available datasets were analyzed in this study. This data can be found here: The Cancer Genome Atlas https://portal.gdc.cancer.gov/Gene Expression Omnibus https://www.ncbi.nlm.nih.gov/geo/The Kaplan Meier plotter http://kmplot.com/analysis/CIBERSORT https://cibersortx.stanford.edu/GDSC www.cancerrxgene.org/.
