# Supplementary material for: Identification of a novel oxidative stress-related prognostic model in lung adenocarcinoma
Source: Front Pharmacol. 2022 Nov 18;13:1030062. doi: 10.3389/fphar.2022.1030062 (PMC9715759; doi:10.3389/fphar.2022.1030062)
Supplement: Supplementary file 1 [file Table1.DOCX]

Table 1

Sequence of primers used in this study.

| Primer name | Primer sequence (5'-3') |
| --- | --- |
| CYP2D6-F | TAGTGGTGGCTGACCTGTTCTCT |
| CYP2D6-R | TCGTCGATCTCCTGTTGGACA |
| CAT-F | CCAGAAGAAAGCGGTCAAGAA |
| CAT-R | GAGATCCGGACTGCACAAAG |
| FMO3-F | AATTCGGGCTGTGATATTGC |
| FMO3-R | TTGAGGAAGGTTCCAAATCG |
| GAPDH-F | GGAGCGAGATCCCTCCAAAAT |
| GAPDH-R | GGCTGTTGTCATACTTCTCATGG |
